# *Cancers* 2025: Growing with Quality

**DOI:** 10.3390/cancers18050853

**Published:** 2026-03-06

**Authors:** Samuel C. Mok

**Affiliations:** Department of Gynecologic Oncology and Reproductive Medicine, The University of Texas MD Anderson Cancer Center, Houston, TX 77030, USA; scmok@mdanderson.org

As we enter 2026, we take a moment to reflect on the progress of *Cancers* over the past year. As a semi-monthly journal publishing 24 issues annually, we continue to serve the global oncology community with open access to cutting-edge research.

In 2025, *Cancers* accounted for 6.75% of publications in Web of Science *Oncology* and 8.81% of the open access oncology category journals, maintaining its position as a leading *Oncology* journal. Our 18 sections cover diverse aspects of cancer research, from the tumor microenvironment to cancer biomarkers, ensuring comprehensive coverage of this rapidly evolving field. We are particularly pleased to note increased submissions from diverse research groups, reflecting our truly global reach. With nearly 75% of papers published within Special Issues, this achievement reflects the success of our Guest Editor program. While our 2024 Journal Impact Factor stands at 4.4 (Five-Year IF: 4.8), placing us in Q2 in the *Oncology* category, we remain focused on quality over quantity. Our CiteScore of 8.8, increasing from 4.3 in 2015 to 8.8 in 2024, places us in Q1 for *Oncology*.

In 2025, *Cancers* actively engaged in global academic exchange through diverse conference initiatives to enhance the journal’s visibility. We hosted our first in-person conference, “Cancers 2025—CancersScape: Spatial Biology of the Tumor Ecosystem,” chaired by Dr. Sammy Ferri-Borgogno, Professor Samuel C. Mok, and Dr. Eduard Porta-Pardo, held in Barcelona, Spain, from 5 to 7 November 2025 [1]. Additionally, we sponsored 75 international conferences, including 15 booths, 10 free media partnerships, 12 paid sponsorships, and 32 travel grants for Editorial Board Members and Guest Editors, covering major events such as the AACR Annual Meeting and EACR Congress. These efforts have strengthened our connections with the global oncology community while providing valuable platforms for scholarly exchange.

Our Editorial Board consists of approximately 2000 leading experts from worldwide institutions including MD Anderson Cancer Center, Memorial Sloan Kettering, Dana-Farber Cancer Institute, German Cancer Research Center (DKFZ), Institut Gustave Roussy, University of Oxford, Karolinska Institutet, and National Cancer Center Japan, uniting global excellence in cancer research. We welcomed 68 new members in 2025 and are grateful to the 509 Board Members who contributed papers and the nearly 800 Board Members actively participating in peer review. We also recognize our 676 Topical Advisory Panel Members and 741 Reviewer Board Members for their essential contributions.

To recognize and support excellence in cancer research, *Cancers* offers a comprehensive awards program designed to celebrate outstanding contributions at various career stages. Our Young Investigator Award (https://www.mdpi.com/journal/cancers/awards/3309, accessed on 4 March 2026, the access dates for the following links are all the same) recognizes and financially supports early career researchers. We also honor emerging scientists through our Best Ph.D. Thesis Award (https://www.mdpi.com/journal/cancers/awards/3611), which accepts applications until 31 May 2026. For our dedicated peer reviewers, the Outstanding Reviewer Award (https://www.mdpi.com/journal/cancers/awards/3319) recognizes those who consistently provide exceptional review quality, with winners to be announced in March 2026. The Best Paper Award (https://www.mdpi.com/journal/cancers/awards/3577) celebrates significant published research, with the winners to be announced on 30 June 2026. Additionally, our Travel Award (https://www.mdpi.com/journal/cancers/awards/3780) program supports researchers attending key conferences, with the application deadline in October 2026, and the Editor of Distinction Award (https://www.mdpi.com/journal/cancers/awards/3702) recognizes the esteemed dedication of editorial board members, with the 2026 winner announcement scheduled for 30 April 2027. These initiatives reflect our journal’s commitment to encouraging outstanding researchers in the field of oncology.

Looking ahead, we will continue prioritizing rigorous peer review and quality support for the dissemination of cancer research. We thank all authors, reviewers, and editors for their trust in *Cancers*. We look forward to advancing oncology science together in 2026.
